# Development of a health care policy characterisation model based on use of private health insurance

**DOI:** 10.1186/1743-8462-2-27

**Published:** 2005-11-08

**Authors:** Rachael E Moorin, C D'Arcy J Holman

**Affiliations:** 1 Australian Centre for Economic Research on Health (ACERH), School of Population Health, The University of Western Australia, Perth, Australia; 2 Centre for Health Services Research, School of Population Health, The University of Western Australia

## Abstract

**Objective:**

The aim of this study was to develop a policy characterisation process based on measuring shifts in use of private health insurance (PHI) immediately following implementation of changes in federal health care policy.

**Method:**

Population-based hospital morbidity data from 1980 to 2001 were used to produce trend lines in the annual proportions of public, privately insured and privately uninsured hospital separations in age-stratified subgroups. A policy characterisation model was developed using visual and statistical assessment of the trend lines associated with changes in federal health care policy.

**Results:**

Of eight changes in federal health care policy, two (introduction of Medicare and Lifetime Health Cover) were directly associated with major changes in the trend lines; however, minor changes in trends were associated with several of the other federal policies. Three types of policy effects were characterised by our model: direction change, magnitude change and inhibition. Results from our model suggest that a policy of Lifetime Health Cover, with a sanction for late adoption of PHI, was immediately successful in changing the private: public mix. The desired effect of the 30% rebate was immediate only in the oldest age group (70+ years), however, introduction of the lifetime health cover and limitations in the model restricted the ability to determine whether or if the rebate had a delayed effect at younger ages.

**Conclusion:**

An outcome-based policy characterisation model is useful in evaluating immediate effects of changes in health care policy.

## Introduction

Private health insurance (PHI) is one of the foundations of the Australian health system [1]. Unlike the Unites States, however, the Australian Government provides universal access to free public hospital care, with ambulatory care and pharmaceuticals being available subject to limited client co-payments via Medicare and the Pharmaceutical Benefits Scheme [2]. The return of a Liberal federal government to Australia in 1996 marked a resurgence of policy interest in the uptake of PHI [3]. The justification for the policies introduced was that falling PHI membership, observed since the introduction of Medicare in 1984, was thought to have increased the demand on the public system [4,5] and, therefore, promoting growth in the private sector would take the pressure off public hospitals and restore balance to the health care system [6]. Subsequent policy initiatives concentrated on increasing PHI coverage by a mixture of 'carrots' (the private health insurance incentive scheme in 1997, partially replaced by a 30% non-means tested rebate on PHI premiums in 1999) and 'sticks' (a Medicare levy surcharge in 1997 for high income earners who did not take out PHI; and Lifetime Health Cover in 2000, whereby higher premiums were paid by those who delayed taking out PHI until after the age of 30 years) [7].

To date, analyses of the effects of policies aimed at supporting PHI in Australia have primarily centred on changes in the proportion of the population covered by PHI [2,4,7,8]. However, a distinction between uptake of PHI and use of PHI is archetypal of the distinction between outputs and outcomes, where outputs document the amount, quality or volume of use of a services product and outcomes reveal the impact the service has on its participants (change in behaviour, attitude or condition) [9]. Changes in the uptake of PHI are necessary but do not of themselves provide sufficient evidence to evaluate outcomes of policies aimed at reducing the pressure on the public system. Rather, the effectiveness of such policies would be better judged by changes in PHI use.

The aim of this study was to use changes in the utilisation of payment classifications for in-patient hospitalisation to develop a process capable of characterising policy changes according to their observable outcomes. The intention being to aid in the analysis of the effects of health care policies directed towards reducing the pressure on the public hospital system.

## Methods

The WA Data Linkage System [10] was used to extract all hospital morbidity data from 1 January 1980 to 31 December 2001 for the State of Western Australia (population 1.8 million), comprising encrypted patient identifiers and episode numbers, age, gender, date of admission, date of separation and payment classification (public, uninsured private, insured private, or "other").

The proportion of the total number of separations in each relevant payment category in each year was calculated according to gender and age group (0–16 yrs, 17–39 yrs, 40–69 yrs, 70+ yrs). The "other" payment categories, which included workers compensation, motor vehicle, defence force personnel and Veteran Affairs patients, were removed from the analysis, leaving only the categories of public, private insured and private uninsured. This was done because the study was principally concerned with elective shifts between private insurance and public categories; not prescribed payment classifications due to mandatory funding arrangements.

### Development of the Policy Characterisation Model

**Figure 1:**
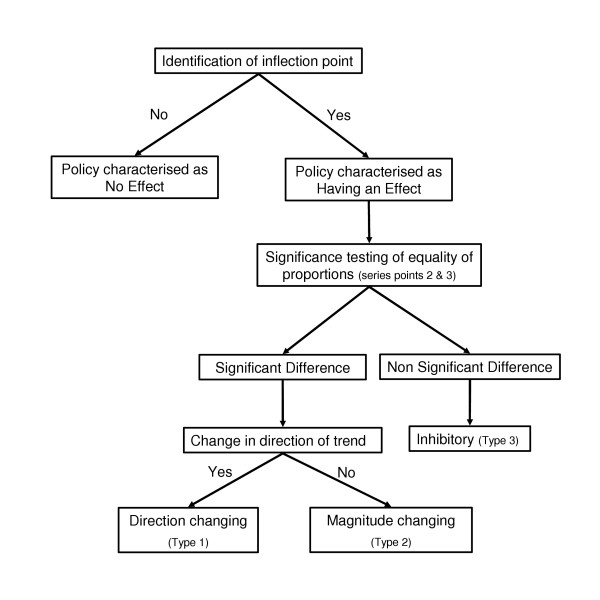
The policy characterisation process.

The annual relative proportion of episodes in each payment classification (public, private insured and private uninsured) were graphed as segmented trend lines stratified by age group and gender. The development of a model to characterise the policies was undertaken by analysing the interaction of the gender and age specific segmented trend lines with the major changes in federal health care policy, termed 'cut points' (see table 1) for each payment classification. The process developed is shown in figure 1 with each component described below.

**Table 1:** Federal health care policy changes (cut points)

**Federal Health Policy "Cut Points"**		
**Cut Point**	Commencement (and duration) of initiative*	**Description of Initiative**
**1**	Sept 1981 (- Jan 1984)	Abolition of free public hospital care
**2**	Feb 1984 (- Oct 1986)	Medicare introduced (Universal bulk billing and free public hospital care restored)
		Out of hospital rebate set at 85% of scheduled fee
		Maximum rebate set at $10
		Levy set at 1%
**3**	Nov 1986 (- June 1993)	Medicare levy increased to 1.25%
		Out of hospital rebate @ 85%/$20
		GAP set at $150/annum
		In hospital rebate set at 75% with no maximum
		Private hospital insurance to cover remaining 25%
**4**	1993 (- 1995)	Medicare Levy increased to 1.4%
**5**	1995 (- 1997)	Medicare Levy increased to 1.5%
		0.2% Surcharge introduced to pay for a guns "buy back" following Port Arthur massacre
**6**	1997 (-1999)	Private Health Insurance Incentive Scheme: Surcharge of 1% introduced for high income household without PHI.
		GAP cover policies allowed (No GAP and known GAP)
		Simplified billing (use of billing agents)
**7**	Jan 1999 (- June 2000)	Uncapped 30% PHI† rebate for hospital and ancillary benefits with no means test
**8**	July 2000 (- Present)	Lifetime Health Cover: Differential premiums allowed based on age at initial premium.
		Informed Consent: Patients provided with quotes on costs prior to procedure commencement

* Financial year unless otherwise indicated

† Private Health Insurance

#### Stage 1: Identification and classification of inflection points in adjacent trend segments

**Figure 2:**
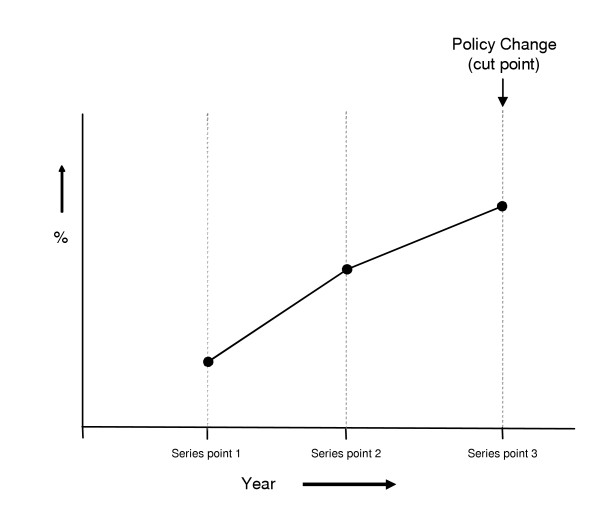
Schematic of the identification of the trend segments included in the analysis.

For each policy change trend segments included in the analysis were determined in the following manner (refer to figure 2):

• Trend segment one was defined as the segmented trend line connecting the proportion of episodes two years prior with that one year prior to the policy change (series points 1 and 2).

• Trend segment two was defined as the segmented trend line connecting the proportion of episodes one year prior with that in the year of implementation of the policy change (series points 2 and 3).

Trend segments one and two were assessed visually to determine the occurrence and classification of inflections (changes in the magnitude or direction of the slope). Inflections were classified as either:

1. Not observed (no appreciable difference in either the magnitude of the slope or direction of trend section two relative to trend section one).

2. Magnitude changing (the slope of trend section two was appreciably different in magnitude to that of trend section one)

3. Direction changing (the direction of trend section two was different to that of trend section one).

Where inflections were not observed, the policy change was deemed to have had no effect on the trend in utilisation and no further analysis was undertaken (refer to figure 1). However, if an inflection was observed the process continued to stage two, as detailed below.

#### Stage 2: Determination of a significant difference in the proportion of episodes

Where an inflection point was identified significance testing of the equality of the proportion of episodes for series points two and three (the year immediately prior to the policy change and the year of implementation of the policy change, refer to figure 2) was performed using a z test based on the normal approximation to the binomial distribution. This test used the z statistic to test the two sided alternative that two proportions were the same.

#### Stage 3: Outcome of the significance testing

Characterisation of those policies deemed to have had an impact was undertaken depending upon the results of the significance testing. A non-significant difference between series points 2 and 3 (p value greater than 0.05) resulted in the policy being deemed as an inhibitory policy (type 3). However, a significant difference between series points 2 and 3 (p value less than or equal to 0.05) required the classification of the direction of the inflection to be integrated into the analysis.

#### Stage 4: Integration of the classification of the inflection

Those policy changes associated with inflections classified by stage one as direction changing were subsequently termed direction changing policies (type 1). While those policy changes associated with inflections classified by stage one as magnitude changing were subsequently termed magnitude changing policies (type 2).

### Quantification of the rates of change associated with inflections

So as to investigate in more detail changes in utilisation associated with observed inflections a separate analysis was conducted quantifying changes in the rate of change of the annual proportion of episodes associated with the introduction of those policies identified in stage 1 as showing an observable inflection. This was achieved by representing each segmented trend segment as a straight line having the following mathematical properties y = a+bx (where 'a' is the intercept and 'b' is the slope). This analysis was carried out for trend segments 1 and 2 (see figure 2). Differences in the rate of change (slope of the trend segment expressed as percentage change per year) for all payment classifications by gender and age group were calculated

## Results

**Figure 3:**
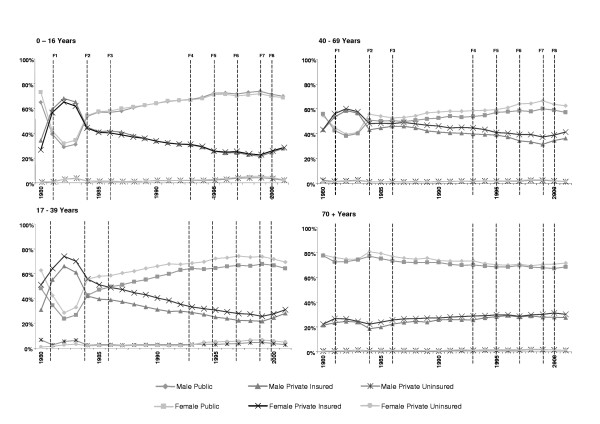
The eight federal cut points overlaid on trend line data for gender and age group.

Figure 3 shows the temporal positions of the eight federal health care policy cut points overlaid on the segmented trend lines of the proportions of annual episodes in each payment classification in each age group in males and females.

### Observation and quantification in changes in trend

In general the shape of the trends was similar in males and females. In some age groups, particularly the 17–39 years age group, there was a near-constant difference in proportion between the genders. Given this finding to simplify the analysis the genders were combined. The shape of the segmented trend lines; however, varied significantly across age groups, with the two younger age groups experiencing the largest changes in payment classification mainly over the early part of the observation period. The oldest age group had the least annual differences and a more stable overall trend.

Federal policy initiatives that were associated with major rate changes or inflections in the trend lines were federal cut points 2 (the re-introduction of free public hospital care via Medicare) and 8 (Lifetime Health Care). Federal cut point 2 was associated with acceleration in the rate of decline in the proportion of privately insured episodes and a greater rate of increase in the proportion of public episodes in all four age groups.

Federal cut point 8 was another major inflection point associated with a surge in the private insurance payment classification in all except the oldest age group. For the younger three age groups the shift in direction was of similar magnitude as shown in table 2. The magnitude of the change in rates associated with the introduction of Lifetime Health Cover was smaller in absolute terms, as well as in the opposite direction to that associated with the introduction of Medicare.

**Table 2:** The rates of change of the proportion of public and private insured episodes pre and post federal cut points 2 (Medicare) and 8 (Lifetime Health Cover).

**Age Group**	**Gender**	**Payment Classification**	**Rate of change in proportion ** (% change in 1 year)		**Change of Direction**	**Difference in Rate **(% change in 1 year)
**Federal cut point 2**			**1982–83**	**1983–84**		

0–16 years	M	Public	2.14	22.45	NO	20.312
	F		2.60	19.73	NO	17.135
	M	Private Insured	-2.99	-20.32	NO	17.333
	F		-3.35	-17.65	NO	14.296
17–39 years	M	Public	4.27	22.82	NO	18.549
	F		3.10	15.83	NO	12.730
	M	Private Insured	-5.17	-18.88	NO	13.709
	F		-3.87	-14.46	NO	10.590
40–69 years	M	Public	1.38	14.87	NO	13.489
	F		1.88	10.52	NO	8.647
	M	Private Insured	-1.99	-13.35	NO	11.362
	F		-2.44	-9.45	NO	7.002
70+ years	M	Public	0.17	5.96	NO	5.797
	F		1.40	2.80	NO	1.401
	M	Private Insured	-0.68	-5.35	NO	4.674
	F		-1.91	-2.28	NO	0.370

**Federal cut point 8**			**1998–99**	**1999–00**		

0–16 years	M	Public	0.90	-2.49	Yes	3.398
	F		0.74	-1.95	Yes	2.691
	M	Private Insured	-1.13	3.09	Yes	4.219
	F		-1.06	3.08	Yes	4.143
17–39 years	M	Public	0.35	-1.85	Yes	2.196
	F		1.44	-1.03	Yes	2.475
	M	Private Insured	-0.43	2.81	Yes	3.232
	F		-1.69	1.98	Yes	3.672
40–69 years	M	Public	2.04	-2.74	Yes	4.787
	F		2.12	-1.01	Yes	3.129
	M	Private Insured	-2.17	3.20	Yes	5.362
	F		-1.97	1.63	Yes	3.608

Less marked changes in the trends, in addition to the major ones described above, were observed to coincide with all federal cut points to some degree, although none was seen consistently in all combinations of payment classification and age group. The largest of these minor rate changes was associated with federal cut point 3 (see table 1) in the youngest age group. These changes involved inflections in the segmented trend lines with absolute differences slightly in excess of 3.5 percent per year. The remaining observable changes ranged from 2.3 percent to 0.7 percent per year.

### Significance testing in those cut points deemed to be associated with inflections

Significance tests of the equivalence of the proportion of episodes one year prior to and in the year of implementation for federal policy cut points associated with observable inflections are summarized in tables 3 and 4. Most federal policy initiatives that showed an observable change in trend were also associated with a significant change (p < 0.05) in private: public mix. The most notable exception to this occurred in the elderly age group. In those aged 70+ years, cut points 7 (designed to increase the proportion of persons holding private health insurance by making it more affordable) was not associated with a significant difference.

**Table 3:** Federal cut points associated with significant (p < 0.05) changes in the proportion of episodes and inflections or substantial changes in trend by age group

**Federal Healthcare Policy Cut Points**			
**Age Group**	**Public**	**Private Insured**	**Private Uninsured**
**Age 0–16 yrs**	2,5,6,8	2,5,8	2
**Age 17–39 yrs**	2,3,5,7,8	2,3,7,8	8
**Age 40 – 69 yrs**	2,4,5,7,8	2,4,5,7,8	2,8
**Age 70+ yrs**	2,6	2,6	

Shaded areas = no cut points associated with significant changes and inflections or trend changes for the age group/couplet type combination

**Table 4:** Federal cut points associated non-significant (p > 0.05) changes in the proportion of episodes and inflections or substantial changes in trend by age group

**Federal Healthcare Policy Cut Points**			
**Age Group**	**Public**	**Private Insured**	**Private Uninsured**
**Age 0–16 yrs**	3	3,6	8
**Age 17–39 yrs**			2,5
**Age 40 – 69 yrs**			
**Age 70+ yrs**	7	7	2

Shaded areas = no cut points associated with non-significant changes and inflections or trend changes for the age group/couplet type combination

The privately uninsured payment classification was the least affected by policy changes over time. However, the two most influential policies, being the introduction of Medicare (cut point 2) and Lifetime Health Cover (cut point 8), were both associated with significant reductions in the proportions of private uninsured patients in several age groups, albeit that the shifts were towards the public and private insured payment classifications respectively. Once again in the oldest age group neither of these cut points was associated with a significant difference.

### Characterisation of policy effects

Four types of policies were identified by the policy characterisation model. Those that had no effect; type 1, those that affected the direction of the trend; type 2, those that affected the magnitude of the trend, but not its direction; and type 3, those that inhibited the trend (the pre policy trend was positive or negative, but significance testing indicated no-significant difference in the proportions post policy). It should be noted that type 3 policies prevented (or subdued) a pre-existing trend from continuing. The results of the characterisation of federal policies from 1980 to 2001 related to age group are detailed in table 5.

**Table 5:** Classification of federal policy effects on trends in age group related annual proportion of episodes by payment classification

**Federal cut point**	**Age Group**											
	**0 – 16 Years**			**17 – 39 Years**			**40 – 69 Years**			**70+ Years**		
	
	**Public**	**Private Insured**	**Private Uninsured**	**Public**	**Private Insured**	**Private Uninsured**	**Public**	**Private Insured**	**Private Uninsured**	**Public**	**Private Insured**	**Private Uninsured**
**F1**	No trend data available prior to 1980*											
**F2**	Type 2	Type 2	Type 2	Type 2	Type 2	Type 3	Type 2	Type 2	Type 2	Type 2	Type 2	Type 3
**F3**	Type 3	Type 3		Type 2	Type 2							
**F4**							Type 1	Type 1				
**F5**	Type 2	Type 2		Type 1		Type 3	Type 2	Type 2				
**F6**	Type 1	Type 3								Type 1	Type 1	
**F7**				Type 1	Type 2		Type 1	Type 2		Type 3	Type 3	
**F8**	Type 1	Type 1	Type 3	Type 1	Type 1	Type 1	Type 1	Type 1	Type 1			

* Since no data was available about the trend 1979–1980 the classification of this policy was not possible. However, in the known historical setting sudden removal of free public hospital care it would be most likely that this policy was a type 1 (direction changing).

Type 1: Direction changing, Type 2: Magnitude changing, Type 3: Inhibitory

Shaded areas indicate no observable effect from the policy change.

## Discussion

In free markets consumers and suppliers are left alone to interact and balance supply and demand for services. It is generally accepted that governments need to intervene in health markets to provide certain services and regulate the market. This intervention occurs via specific policy action [11]. In Australia the Commonwealth Government's decision to subsidise PHI has meant that it has increased its stake in the private sector alongside its existing stake in the public sector.

Controversy has raged about the success of the Commonwealth Government's policies with regard to supporting PHI in order to reduce the pressure on the public sector. The major debate has centered around the effectiveness of the 30% rebate and more recently the effectiveness of the Lifetime Health Care policy [4,7,2-15]. However, in most cases, commentators have used evidence relating to the changing prevalence of PHI membership, pre and post policy implementation. This may not be an accurate method to assess the effectiveness of such policies, because the policies themselves may promote the uptake of PHI for non-health related reasons, such as to avoid a tax penalty in high income households (cut point 6). This coupled with the finding that since 1998 the proportion of PHI fund members with high front-end deductibles has significantly increased [4] means that uptake of PHI may not necessarily lead to the expected changes in use of the public and private systems. This is quite apart from the debate about the price elasticity of demand for PHI and the assumption that demand for hospital care is a fixed commodity [4]. Our study has developed and used a policy characterisation model based on measuring shifts in the actual use of PHI at the time of receiving hospital services. This may be a more appropriate methodology for evaluating likely changes in the pressure on the public system affected by particular policies.

The results of our analysis indicate that federal cut point 2, the re-introduction of free public hospital care via Medicare, was a magnitude changing policy. This was an unexpected finding since it has been previously assumed that the introduction of Medicare, following on from an era when free public hospital care was abolished, would be a direction changing policy. However, our data indicate that a reversal in trend in favour of the public system occurred one year prior to the introduction of Medicare. Federal cut point 8, Lifetime Health Cover, was classified by our model as a direction changing policy in the younger three age groups with no effect observed in the oldest age group (individuals born prior to 1 July 1934 are exempt from Lifetime Health Cover). This finding was thus consistent with the objective of the policy, which was to reverse the declining trend in possession and use of PHI to reduce the burden on the public system. It would appear that this was achieved immediately post-implementation.

The effects of the 30% rebate (federal cut point 7) on levels of PHI have been one of the most hotly contested political issues surrounding heath care policy in recent times. Commentators have argued for and against this policy initiative mainly on a cost-benefit platform [4, 12, 14, 16]. Our analysis found that the effect of federal cut point 7 was related to age. This policy was associated immediately in time with a change in the magnitude of the existing negative trend (PHI) or a negative to positive change of direction (public) in the middle two age groups, and an inhibitory effect on the downward trend in the oldest age group, with no effect observed in the youngest age group. Thus the 30% rebate appears to have had the desired effect on PHI use (ie reducing the pressure on the public system) in the oldest age group, but no immediate desired effect in the younger age groups. To some extent this can be explained because younger members are not as likely to be hospitalised compared with older members, thus reducing the likelihood of an immediate effect on use. While older Australians are not only more likely to be hospitalised and therefore have more opportunity to use PHI, but are also more likely to be attracted to purchase PHI due to reduced cost because the price elasticity of PHI is different for younger and older individuals.

### Limitations of the model

For practical reasons the immediate effects of the policies were the only effects able to be characterised by our model due to the plethora of federal health care policy changes, especially from 1993 onwards. To try to characterise the changes in trends over an extended period would have resulted in evaluation of the mix of effects produced by more than one policy. This is especially relevant when examining the effects of the 30% rebate and Lifetime Health Cover, where only one year separated the two policy changes.

A second limitation of this policy characterisation method is that it cannot accurately take into account enforced waiting periods, which are mandatorily applied to individuals taking out PHI who have a previous history of an illness or condition. Thus some underestimation of the effects of policies may be inherent in the model. Extending the model over two years post policy initiation is problematic, as discussed above, because the effect observed would then be confounded by subsequent policy changes. Another issue to be taken into account is the timing and extent of marketing of the policy to the public by government and the private insurance industry. In the case of Lifetime Health Cover, exhaustive marketing, the 'Run for Cover Campaign', was undertaken over several months leading up to its implementation. It is reasonable to assume that changes in behaviour, in this case purchasing of PHI, were likely to have been made prior to the policy implementation date, thus some of the waiting period, if applicable, would have been served prior to the policy implementation date. Conversely, advantage could be taken of the 30% rebate at any time after, but not before January 1999. The net result of these two limitations on our policy characterisation model may be that of cancelling each other out in the case of Lifetime Health Cover and causing a latent period between cause and effect in the case of the 30% rebate.

In addition, it could be argued that since the waiting period only applies to pre-existing conditions, those wishing to use newly acquired PHI for such a condition would be doing so to facilitate a more expedient health intervention than could be achieved in the public sector. As such these episodes of care would not normally have been observed in the public system over the same period, but rather at a later time. Under these circumstances the waiting time for benefits may serve to enhance the validity of a characterisation model employing a latent period.

Finally, our policy characterisation model does not allow for the possibility of an earlier policy initiative synergising with a subsequent initiative. Thus it is possible that the immediate effect of Lifetime Health Cover may have been less potent in the absence of the pre-existing 30% rebate.

## Conclusion

Our study has developed and applied a policy characterisation model based on measuring shifts in use of PHI immediately prior to, and immediately following implementation of changes in federal health care policy. Our results indicate that Lifetime Health Cover was associated with an immediate increase in patients in hospital using PHI. While the 30% rebate for PHI introduced 18 months earlier did not have an immediate desired effect, the limitations of the model are such that we cannot be certain what, if any, latent contribution to the change in private: public mix may have occurred. From this study we conclude that an outcome-based policy characterisation model is useful in evaluating immediate effects of changes in health care policy.

## Competing interests

Professor D'Arcy Holman is an independent director of HBF Health Funds inc which is the largest provider of private health insurance in Western Australia.

## Authors' contributions

The manuscript has been read and approved by all authors and the requirements for authorship have been met as outlined below. REM was responsible for the conception and design of the study; analysis and interpretation of the data; and drafting and revising the paper. CDJH was responsible for conception and design of the study; interpretation of the data; and revising the paper.

## References

[B1] Sullivan N, Redpath R, O'Donnell A (2002). Public Hospitals: Who's looking after you? The Difficulties in Encouraging Patients to use their Private Health Insurance in Public Hospitals. Australian Health Review.

[B2] Willcox S (2001). Promoting Private Health Insurance in Australia. Health Affairs.

[B3] Duckett SJ (2000). The Australian Health Care System.

[B4] Deeble J (2003). The Private Health Insurance Rebate: Report to State and Territory Health Ministers.

[B5] McAuley IA (2004). Stress on public hospitals - why private insurance has made it worse.

[B6] Duckett SJ, Jackson TJ (2000). The New health Insurance Rebate: An Inefficient Way of Assisting Public Hospitals. Medical Journal of Australia.

[B7] Butler J (2002). Policy Change and Private Health Insurance: Did the Cheapest Policy do the Trick?. Australian Health Review.

[B8] Cormack M (2002). Private Health Insurance: The Problem Child Faces Adulthood. Australian Health Review.

[B9] National Network of Libraries of Medicine Define Measurable Goals, Outputs and Outcomes. http://nnlm.gov/libinfo/community/goals.php.

[B10] Holman CDJ, Bass AJ, Rouse IL, Hobbs MST (1999). Western Australia: Development of a Health Services Research Linked Database. Aust NZ J Public Health.

[B11] Whiteford H (2001). Can Research Influence Mental Health Policy?. Australian and New Zealand Journal of Psychiatry.

[B12] Australian Health Insurance Association (2003). AHIA Submission on PHI Reforms to Senate Legislation Committee: Health Legislation Amendment (Private Health Insurance Reform) Bill 2003.

[B13] Harper I (2003). Preserving ChoiceA defence of public support for private health care funding in Australia. Medibank Private.

[B14] Hagan P, Econtech Pty Ltd, Harper Associates (2004). Easing the Pressure: The Intergenerational Report and Private Health Insurance.

[B15] Access Economics (2002). Striking a Balance: Choice, Access and Affordability in Australian Health Care.

[B16] Segal L (2004). Why it is time to review the role of private health insurance in Australia. Australian Health Review.

